# Advancements in Optical Fiber Sensors for pH Measurement: Technologies and Applications

**DOI:** 10.3390/s25144275

**Published:** 2025-07-09

**Authors:** Alaa N. D. Alhussein, Mohammed R. T. M. Qaid, Timur Agliullin, Bulat Valeev, Oleg Morozov, Airat Sakhabutdinov, Yuri A. Konstantinov

**Affiliations:** 1 Department of Radiophotonics and Microwave Technologies, Kazan National Research Technical University Named After A. N. Tupolev-KAI, 10, K. Marx St., Kazan 420111, Russia; alkhusseyna@stud.kai.ru (A.N.D.A.); kaidmr@stud.kai.ru (M.R.T.M.Q.); taagliullin@kai.ru (T.A.); bivaleev@kai.ru (B.V.); azhsakhabutdinov@kai.ru (A.S.); 2 Perm Federal Research Center of the Ural Branch of the Russian Academy of Sciences (PFRC UB RAS), 13a Lenin St., Perm 614000, Russia; yuri.al.konstantinov@ro.ru

**Keywords:** optical fiber sensors, pH measurement, evanescent wave, SPR and FBG sensors, real-time monitoring, bio-medical and industrial applications

## Abstract

Measuring pH is a critical parameter in environmental monitoring, biomedical diagnostics, food safety, and industrial processes. Optical fiber sensors have proven highly effective for pH detection due to their exceptional sensitivity, rapid response, and resistance to electromagnetic interference, making them well suited for real-time monitoring. This review offers a comprehensive analysis of recent advances in optical fiber-based pH sensors, covering key techniques such as fluorescence-based, absorbance-based, evanescent wave, and interferometric methods. Innovations in Fiber Bragg Grating and Surface Plasmon Resonance technologies are also examined. The discussion extends to the impact of pH-sensitive coatings—ranging from nanomaterials and polymeric films to graphene-based compounds—on enhancing sensor performance. Recent advancements have also enabled automation in data analysis and improvements in remote sensing capabilities. The review further compares the economic viability of optical fiber sensors with traditional electrochemical methods, while acknowledging persistent issues such as temperature cross-sensitivity, long-term stability, and fabrication costs. Overall, recent developments have broadened the functionality and application scope of these sensors by improving efficiency, accuracy, and scalability. Future research directions are outlined, including advanced optical interrogation techniques, such as Addressed Fiber Bragg Structures (AFBSs), microwave photonic integration, and optimized material selection. These approaches aim to enhance performance, reduce costs, and enable the broader adoption of optical fiber pH sensors.

## 1. Introduction

Acids and bases are essential chemical entities significantly involved in diverse biological, environmental, and industrial processes. Investigating their characteristics is fundamental to both theoretical research and practical applications [1,2]. Chemical substances are classified as acids, bases, or neutral compounds based primarily on their behavior in aqueous solutions and corresponding pH values [3,4]. Furthermore, acids and bases can be categorized as strong or weak based on their degree of ionization and reactivity. The precise measurement of pH, indicative of solution acidity or alkalinity, is crucial across various fields including chemical analysis, environmental monitoring, and medical diagnostics [5]. Consequently, numerous methodologies, each based on distinct physical and chemical principles, have been developed for pH determination [6,7]. These methods differ significantly regarding precision, sensitivity, operational costs, and suitability for particular applications. For instance, glass electrodes exhibit high accuracy, ease of use, and a wide pH measurement range, but their fragility and requirement of frequent calibration limit their utility in harsh environments [8,9,10,11]. Solid-state electrodes, while robust and suitable for harsh environments, typically exhibit lower sensitivity and accuracy [12,13,14]. Ion-selective field-effect transistor (ISFET) sensors offer durability, portability, and versatility across diverse environments, but are accompanied by higher costs and complex electronics [15,16,17]. Colorimetric indicators represent a simple, rapid, and cost-effective method; however, their reliance on visual estimation reduces accuracy significantly [18,19,20]. The demand for robust, high-sensitivity, and real-time monitoring solutions has driven interest in optical fiber sensors. These sensors exhibit high accuracy, rapid response, and immunity to electromagnetic interference, making them particularly suitable for harsh or complex conditions [21,22,23,24]. Among recent innovations, fluorescence- and absorbance-based optical fiber sensors have emerged as reliable alternatives. Their integration with advanced technologies, such as Fiber Bragg Grating (FBG) and Surface Plasmon Resonance (SPR), has greatly expanded their utility and precision in both biomedical and industrial applications [25,26,27,28,29]. These advances have broadened the functional scope of pH measurement technologies by enhancing selectivity, reducing cross-sensitivities (such as to temperature), and enabling miniaturization and remote sensing.

Their versatility renders them indispensable in areas where they deliver reliable performance even under complex conditions. Recent advances, including the integration of FBG and SPR technologies, have significantly improved the accuracy and functionality of these systems. Such innovations effectively address issues like thermal cross-sensitivity and broaden the operational range of optical fiber sensors in challenging environments.

This review provides a comprehensive analysis of optical fiber-based pH sensing technologies by examining their underlying principles, design characteristics, and practical applications. A common characteristic of all fiber-optic sensors is their versatility and suitability for applications across various fields, including chemical analysis, environmental monitoring, aerospace, and medical diagnostics. This is achieved through the non-invasive nature of the employed methods, as well as the complete electromagnetic and biological neutrality of optical fibers. By delineating their advantages, limitations, and prospective developments, the study emphasizes the transformative potential of these sensors in advancing pH measurement across both scientific and industrial fields.

## 2. Comparative Summary of Electrochemical and Optical pH Sensors

The selection of an appropriate pH sensing method depends on various factors including measurement accuracy, application environment, response time, and cost. Traditional electrochemical techniques such as glass electrodes and ISFET sensors are widely used due to their simplicity and reliability; however, they often face limitations in harsh or remote settings. Optical fiber-based and nanomaterial-enhanced sensors have recently gained prominence due to their miniaturization, biocompatibility, and suitability for real-time, distributed measurements. Table 1 presents a comparative overview of key pH sensing methods—highlighting their operational advantages, limitations, and cost implications—to contextualize the need for advanced optical fiber-based solutions.

## 3. Optical Fiber Methods for pH Measurement

Optical fiber-based techniques have emerged as highly sensitive, non-invasive solutions for pH sensing, thereby broadening their utility in diverse fields. This review categorizes optical fiber pH sensors into six primary types—fluorescence-based, absorbance-based, Surface Plasmon Resonance (SPR), Fiber Bragg Grating (FBG), interferometric, and evanescent wave sensors—based on their underlying optical transduction mechanisms. This classification reflects the dominant structure in the recent literature and is used with the intention of providing a clear and systematic comparison. While some overlap may exist in hybrid systems, this approach facilitates an organized understanding of each sensing principle and its distinct operational features. Advanced detection methods include fluorescence-based sensors [22,34,35], absorbance-based optical fiber sensors (OFSs) [21,36,37], SPR-based sensors [38,39,40], interferometric sensors [41,42,43], FBG-based sensors [44,45], luminescence lifetime-based sensors [46,47], and optical fiber evanescent wave sensors (OFEWSs) [48].

These methodologies offer significant advantages, including a broad dynamic measurement range. In certain configurations, optical fiber-based pH sensors can deliver higher accuracy than traditional commercial electrodes. Moreover, specialized designs optimized for specific environments—such as marine microenvironments [21]—have demonstrated exceptional performance. Their inherent adaptability facilitates the development of miniaturized, portable systems, enabling seamless integration with advanced platforms, including smartphone-based detection systems [34]. The explorations into the fundamental light–matter interaction mechanisms have provided deeper insights into the functionality of these sensors. Specifically, the interactions between incident light and the molecular structures within pH-sensitive materials induce measurable shifts in wavelength or intensity variations. This allows for highly accurate measurements even in complex environmental conditions [49,50].

Table 2 presents a comprehensive comparative analysis of various optical fiber sensor technologies, detailing their operating principles, materials, detection mechanisms, applications, advantages, and limitations. This comparison highlights critical performance indicators—such as sensitivity, long-term stability, real-time monitoring capabilities, and cost-effectiveness—thereby guiding researchers and engineers in selecting the most appropriate sensing technology for specific applications.

The special properties of fiber-optic sensors make them particularly suitable for applications requiring high measurement accuracy, such as in aerospace, industrial processes, and advanced biomedical fields [51]. The established relationship between optical parameters—including attenuation, absorption, and scattering—and pH variations underlines their potential to deliver exceptional accuracy even under challenging operating conditions [52].

Despite their many advantages, fiber-optic pH sensors still face certain limitations in terms of material durability, environmental stability, and manufacturing costs. Overcoming these challenges through scaled-up manufacturing processes and robust material development has the potential to significantly reduce costs, thereby improving affordability and encouraging wider adoption.

**Table 2:** A comparison of optical fiber-based pH sensor technologies, detailing their operating principles, functional roles, constituent materials, light sources, detection mechanisms, signal processing methods, advantages, and associated challenges.

Aspect	Fluorescence-Based Optical Fiber Sensors	Absorbance-Based Optical Fiber Sensors	SPR-Based Fiber Sensors	Interferometric Fiber Sensors	FBG Sensors	Luminescence Lifetime-Based Sensors	Optical Fiber Evanescent Wave Sensors
Principle	Fluorescence emission [53].	Light absorption [54,55].	Refractive index change [56,57].	Light interference [58].	Wavelength shift [59].	Decay time measurement [60].	Evanescent waves [61,62].
Fiber Role	Guides excitation/collection [63].	Light transmission [64].	Guides and collects light [65].	Interference medium [66].	Sensing/light guide [67].	Guides and collects light [68].	Analyte interaction [69].
Material	Fluorophores/dyes [70].	Coated/uncoated fibers [71].	Gold/silver [72].	Silica/polymers [73].	Refractive index-modulated silica [74].	Luminescent dyes [75].	Silica/polymers [76].
Light Source	Lasers/LEDs [77].	LEDs/lasers [78].	Polarized lasers [79].	Laser diode/LED [80].	Narrowband lasers [81].	Pulsed lasers/LEDs [82].	Lasers/LEDs [83].
Detection Mechanism	Intensity/wavelength shifts [84].	Intensity changes [85].	Resonance shifts [86].	Phase/intensity changes [87].	Wavelength shifts [88].	Decay time analysis [89].	Signal variation [90].
Detectors	Photodiodes/spectrometers [91].	Photodiodes/spectrometers [62].	CCD/CMOS [92].	Photodiodes [93].	Spectrum analyzers [94].	Photomultipliers [95].	Photodiodes [96].
Signal Processing	Fluorescence analysis [97].	Absorbance quantification [98].	Resonance analysis [99].	Interference data [100].	Wavelength conversion [101].	Decay time correlation [102].	Signal variation analysis [103].
Applications	Biomedical, environment [104,105].	Environment, industry [106].	Diagnostics, monitoring [107,108].	Structural, medical [109].	Structural, aerospace [110].	Diagnostics, control [111].	Chemical, biosensing [48].
Advantages	Sensitive, real-time [112].	Compact, remote [113].	Real-time, label-free [114].	EMI immunity, sensitive [115].	Multiplexing, sensitive [116].	Intensity stability [117].	Compact, real-time [118].
Challenges	Photobleaching, cost [119].	Cross-sensitivity [120].	Noise, fouling [121].	Noise, cost [122].	Expensive systems [123].	High cost, quenching [124].	Noise, fabrication [125].

### 3.1. Fluorescence-Based Optical Fiber Sensors

Fluorescence-based optical fiber sensors rely on the emission of light by fluorophores upon excitation at specific wavelengths [126]. In pH sensing, these fluorophores exhibit protonation-dependent spectral shifts in either intensity or emission wavelength, providing a direct optical signal of hydrogen ion concentration. The sensing mechanism typically involves intensity-based fluorescence, where the signal magnitude varies with pH, or lifetime-based fluorescence, where the fluorescence decay time is modulated by the surrounding pH. A pH-sensitive fluorescent dye—such as fluorescein, rhodamine, or carboxyfluorescein—is immobilized on the fiber tip or within a hydrogel matrix, enabling remote and real-time monitoring with high spatial resolution. These systems are valued for their high sensitivity, rapid response, and resistance to electromagnetic interference. Fluorescence-based optical fiber sensors have been demonstrated to be highly effective tools for pH measuring. These sensors rely on the use of fluorescent dyes, which undergo a change in emission intensity or wavelength in response to pH variations. The immobilization of these dyes, whether at the fiber tip or within the sensing region, is pivotal in ensuring high sensitivity and accuracy. Beyond the scope of pH sensing, these sensors find application in temperature monitoring and metal ion detection [127].

#### 3.1.1. Materials and Design Features

Various fluorescent materials are employed to enhance sensor performance, including rare earth polymer composites (e.g., europium-based coordination polymers for heavy metal detection), biomaterials (such as mApple fluorescent protein-based biosensors for cadmium detection), and luminescent nanomaterials (for instance, CdSe/ZnS quantum dots conjugated with pH-sensitive proteins for acidity measurement) [128]. Notably, CdSe/ZnS quantum dots—classified as nanomaterials due to their nanocrystal sizes ranging from 2 to 10 nm—exhibit high photostability, narrow fluorescence spectra, and a broad absorption range. These characteristics significantly improve the sensitivity and stability of the emitted signal, rendering them ideal for optical sensors utilizing ratiometric methods.

Recent advancements have integrated optical fiber sensors with plasmonic sensing systems, resulting in reduced power consumption, enhanced remote sensing capabilities, and simplified configurations [129]. Moreover, the use of photonic crystal fibers further augments detection precision by extending the interaction length between light and the measurement medium, thereby enabling the detection of trace metal ions and biomolecules even in minimal sample volumes [104,127].

#### 3.1.2. Measurement Range

Fluorescence-based optical fiber sensors can achieve broad pH measurement ranges by employing ratiometric techniques and optimized fluorophore combinations. For instance, a sensor utilizing CdSe/ZnS quantum dots and Oxazine 170 embedded in ethyl cellulose achieved reliable detection across pH 1.6–13.2, enabled by a stable Förster Resonance Energy Transfer (FRET) mechanism between dual emission peaks at 575 nm and 655 nm [130]. Similarly, a microfluidic-integrated sensor using HPTS dye demonstrated linear and reproducible measurements within the pH 2.5–9.0 range, suitable for biological and environmental monitoring [131]. For alkaline environments, a sensor based on coumarin–imidazole dye effectively covered the pH 10.0–13.2 range, proving useful in corrosion monitoring applications [132].

These studies confirm that fluorescence-based optical fiber sensors can reliably cover the full practical pH spectrum, from strongly acidic to highly alkaline conditions, with high sensitivity and stability.

#### 3.1.3. Sensitivity

The exceptional sensitivity of these sensors allows for the detection of even minimal pH fluctuations, with the highest reported sensitivity reaching 0.02 pH units via dual-lifetime referencing (t-DLR) techniques [133]. This level of precision renders fluorescence-based sensors highly valuable for monitoring pH in microenvironments, including both biological and marine ecosystems. The incorporation of CdSe/ZnS quantum dots further enhances the fluorescence intensity, thereby improving the signal-to-noise ratio and measurement accuracy [134,135]. Nonetheless, despite their high sensitivity, rapid response, and broad applicability, significant challenges persist in developing sensor designs that are both cost-effective and durable, while also offering high selectivity for practical applications [34]. Current research efforts are directed towards optimizing fabrication methods and enhancing sensor stability to address these limitations [127].

#### 3.1.4. Advantages, Challenges, and Recent Developments

Fluorescence-based optical fiber sensors present numerous advantages, such as rapid response times, high sensitivity, and broad applicability for detecting a variety of substances. Typical fluorescence-based pH sensors operate within a pH range of 1.6–13.2, with sensitivities of around 0.02 pH units and response times as short as 5–10 s, depending on the fluorophore system and integration technique [133]. Nevertheless, challenges remain in developing cost-effective and durable sensor designs that exhibit enhanced selectivity—that is, the capacity to accurately discern specific target substances within complex analyte mixtures, especially in challenging solution matrices and suspensions.

For example, these sensors can be optimized to detect heavy metal ions (Pb^2+^, Hg^2+^, Fe^3+^) in aqueous solutions, volatile organic compounds (VOCs) in industrial emissions, and biomolecules such as glucose or proteins in biological fluids. High selectivity is attained by incorporating specialized fluorophores, molecular receptors, or nanostructured coatings that facilitate specific binding with target analytes while minimizing interference from other environmental components.

To overcome these challenges, researchers are exploring novel technologies, such as the integration of nanostructured coatings, graphene, and molecular receptors, which improve detection precision while minimizing interference from extraneous substances. Advances in materials science—exemplified by the incorporation of graphene and other nanostructured coatings—offer promising solutions by enhancing both the durability and specificity of these sensors.

Recent studies indicate that graphene-based coatings substantially improve both the chemical and mechanical stability of sensors, thereby mitigating degradation in harsh environments [136]. Furthermore, nanostructured layers enhance the selective binding of target molecules, a finding supported by multiple experimental investigations [137]. These advancements have been validated in applications for detecting heavy metals and organic compounds in complex matrices [138].

Current research efforts are dedicated to optimizing sensor fabrication techniques to broaden their applicability across diverse fields. This includes the integration of novel nanomaterials, the enhancement of durability in harsh environments, and the development of more selective sensor elements tailored for specific analytical applications, such as, for example, water quality control [127,135].

**Figure 1:**
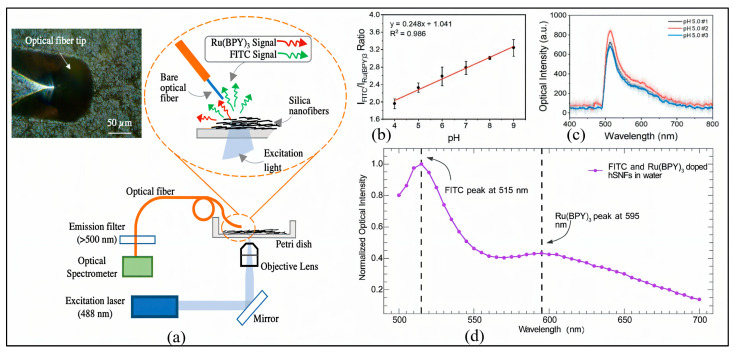
(**a**) Schematic of the fiber-optic pH sensing setup integrating dual-emission hSNFs excited at 488 nm. (**b**) Linear correlation between the FITC/Ru(BPY)_3_ fluorescence ratio and pH (R^2^ = 0.986) over the range 4–9. (**c**) Emission spectra at pH 5.0, 5.5, and 6.0, showing pH-dependent fluorescence intensity shifts. (**d**) Normalized dual-emission peaks at 515 nm (FITC) and 595 nm (Ru(BPY)_3_), enabling ratiometric sensing [139].

In parallel, recent developments have introduced advanced fluorescence-based platforms for real-time pH monitoring [139]. As demonstrated in Figure 1a, an advanced fiber-optic setup was engineered by integrating dual fluorescent dyes—fluorescein isothiocyanate (FITC) as a pH-sensitive indicator and tris(2,2′-bipyridyl)ruthenium(II) chloride (Ru(BPY)_3_) as a reference—within hollow silica nanofibers (hSNFs). The system utilizes near-field excitation with a 488 nm laser and fiber-coupled detection to enable localized fluorescence acquisition.

The ratiometric response shown in Figure 1b reveals a strong linear correlation (R^2^ = 0.986) between the FITC/Ru(BPY)_3_ intensity ratio and pH values from 4 to 9, confirming high analytical precision in aqueous conditions. Figure 1c further demonstrates spectral variations at pH 5.0, 5.5, and 6.0, indicating suitability for physiological monitoring. The distinct emission peaks at 515 nm and 595 nm, highlighted in Figure 1d, form the core of the ratiometric sensing mechanism, enabling reproducible, stable, and real-time measurements.

In a complementary strategy, neutral red (NR) dye embedded in poly(HEMA) hydrogel has been applied to optical fiber tips or sleeves, enabling rapid detection (response time ~5 s) across the pH range 5–8, with a sensitivity of up to 17 nm/pH. These sensors proved effective in simulated biological environments, reinforcing their potential for biomedical diagnostics and environmental pH sensing.

### 3.2. Absorbance-Based Optical Fiber Sensors: Materials, Design, and Performance

Absorbance-based optical fiber sensors represent a reliable and versatile approach for pH monitoring. Their operation is based on the use of indicator dyes whose optical absorbance spectra change in response to the hydrogen ion concentration. These dyes are typically immobilized within pH-sensitive coatings or hydrogels applied to the fiber surface, where changes in the protonation state lead to measurable variations in light attenuation at specific wavelengths. The sensor’s performance is influenced by several design factors, including the choice of dye, coating thickness, and fiber geometry, all of which affect the sensitivity, dynamic range, and response time.

Despite their simplicity and potential for miniaturization, absorbance-based sensors face challenges such as dye leaching, photobleaching, and limited pH detection ranges. These limitations can compromise long-term stability and measurement repeatability, especially in complex environments. Ongoing research focuses on improving the durability of sensing layers, enhancing optical stability, and expanding the effective pH range through novel materials and encapsulation strategies. These efforts aim to make absorbance-based fiber sensors more robust and suitable for continuous, real-time pH monitoring in diverse applications.

#### 3.2.1. Materials and Design Features

**Figure 2:**
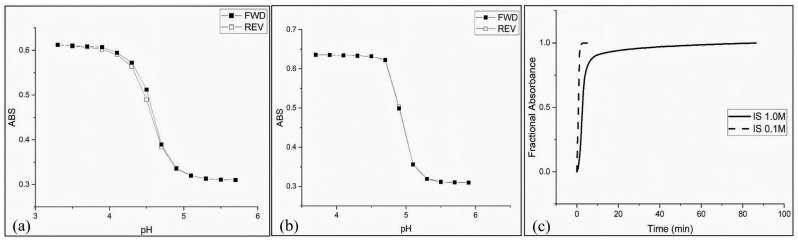
Optical response of the M-70 hydrogel-based sensing layer to pH and ionic strength variations. (**a**,**b**) Absorbance profiles during forward (FWD) and reverse (REV) pH sweeps at 23.5 °C, demonstrating reversible optical transitions at buffer concentrations of 50 mM and 5 mM, respectively. (**c**) Kinetics of polymer shrinking upon acidification (pH 6.1 to 3.3) at high (1.0 M) and moderate (0.1 M) ionic strengths, illustrating the influence of ionic environment on response time [140].

These sensor types employ innovative designs that replace the conventional outer cladding with a sensitive layer. These layers, composed of functional materials such as titanium dioxide (TiO_2_) and silicon dioxide (SiO_2_), offer high thermal and chemical stability while substantially enhancing sensor sensitivity [36]. In recent developments, polymer-based hydrogel layers—particularly those incorporating pH-responsive colloidal microspheres such as poly(N-isopropylacrylamide-co-methacrylic acid)—have been explored as sensitive coatings [140]. These layers demonstrate reversible swelling and shrinking behavior upon pH changes, modulating light absorbance due to changes in their internal refractive index distribution. Figure 2 illustrates the absorbance response of the M-70 copolymer hydrogel system under various conditions: (a) and (b) show forward (FWD) and reverse (REV) pH response profiles at different buffer concentrations (50 mM and 5 mM, respectively), highlighting the sensor’s dynamic equilibrium behavior. (c) presents the time-resolved fractional absorbance change under ionic strengths of 1.0 M and 0.1 M, demonstrating the influence of the ionic environment on polymer shrinking kinetics.

These results underscore the importance of optimizing material formulation and environmental conditions to achieve rapid and reliable pH-sensitive optical responses in absorbance-based fiber sensors. Additionally, these layers are integrated with nanostructures such as zinc oxide (ZnO), thereby enhancing the sensor’s responsiveness to environmental changes. The incorporation of nanomaterials, including gold nanoparticles, represents a significant advancement, increasing the sensitivity and accuracy of these sensors by up to ten times compared to the traditional techniques [141,142].

#### 3.2.2. Measurement Range

Under standard conditions, absorbance-based optical fiber sensors function effectively within a pH range of 4 to 10, where common sensing layers exhibit optimal ionization [36]. However, this range can be extended by engineering the sensor coating—particularly through the incorporation of weak acids with tailored pKa values, such as acrylic acid (AA) or propylacrylic acid (PAA) [140]. For example, poly(N-isopropylacrylamide)-based hydrogels have demonstrated reliable optical responses below pH 3 and above pH 11, depending on the polymer composition, cross-linking density, and environmental factors such as ionic strength and temperature. These strategies allow for sensor adaptation to extreme conditions, including seawater monitoring and industrial effluent analysis [142].

#### 3.2.3. Sensitivity

Absorbance-based optical fiber sensors exhibit exceptional sensitivity, enabling the precise detection of minor fluctuations in analyte concentrations, including pH. For instance, no-core fiber designs relying on wavelength shift measurements have demonstrated sensitivities reaching approximately 0.44 nm/pH, highlighting their high accuracy in detecting subtle changes in pH [37]. Additionally, incorporating advanced nanocoatings has significantly improved the detection limits of these sensors, allowing for the detection of analyte concentrations as low as 10 μM for solutions such as FeCl_3_ [143]. Furthermore, these sensors provide rapid response times (~10 s), making them highly suitable for real-time monitoring applications [36].

#### 3.2.4. Advantages, Challenges, and Recent Developments

Absorbance-based optical fiber sensors offer several key advantages, rendering them highly valuable for a wide range of applications. Their enhanced precision—supported by nanocoating technologies—enables the accurate detection of subtle pH variations and other analytes [143].

Typically, absorbance-based optical fiber pH sensors operate within a pH range of 4–10 (with some designs extending from 3 to 11), exhibit sensitivities around 0.44 nm/pH, and achieve response times of approximately 10 s [37].

Moreover, their ability to function under harsh conditions, including high temperatures and rapid pH fluctuations (response time ~10 s), ensures long-term stability and robustness [37]. Their miniaturized format also facilitates their integration into compact and portable systems across medical and industrial domains [142].

Despite these advantages, challenges persist. High temperatures can cause material expansion and dye leaching, which compromise performance [37]. Dye instability, in particular, remains a key limitation, necessitating the development of more durable coatings [142]. Additionally, cross-sensitivity to interfering substances can impair measurement accuracy, highlighting the need for functional layers that enhance selectivity [36].

To address these limitations, recent advances have focused on improving material stability, sensitivity, and environmental resilience. Incorporating nanostructures such as zinc oxide (ZnO) has improved responsiveness to minor analyte fluctuations [141]. Hybrid coatings that combine polymers with inorganic elements like gold nanoparticles have shown increased mechanical strength and long-term accuracy [142]. Furthermore, advanced fabrication techniques—such as nano-etching and precision deposition—have enabled enhanced signal quality even at low analyte concentrations. The integration of self-referencing mechanisms has also minimized errors from environmental interference (e.g., temperature, humidity, electromagnetic fields, and mechanical vibration), ensuring consistent performance under complex conditions [21].

### 3.3. Surface Plasmon Resonance Sensors

SPR-based optical fiber sensors utilize the resonant oscillation of conduction electrons at the interface between a metal film (typically gold or silver) and a dielectric material. In pH sensing applications, a pH-sensitive recognition layer is deposited onto the metal-coated fiber surface. Changes in pH modify the refractive index of this layer, thereby shifting the resonance condition of the surface plasmons. This shift is detected as a change in the intensity or wavelength of the reflected light. SPR sensors offer high sensitivity and are especially suitable for detecting minute changes in surface-bound analyte concentrations under aqueous conditions.

SPR-based sensors have garnered considerable attention for their ability to perform real-time, label-free detection without the need for fluorescent, radioactive, or chemical markers. This capability enables the direct monitoring of molecular interactions through the detection of refractive index changes at the sensor surface, thereby ensuring exceptional sensitivity [144]. Moreover, recent technological advancements—such as SPR imaging (SPRI) and electrochemiluminescent (ECL)-enhanced biosensors—have further broadened the application scope of SPR-based platforms. This mechanism has been effectively adapted for pH sensing by correlating shifts in resonance conditions with proton-induced refractive index changes [145].

A notable example is the Tilted Fiber Bragg Grating–Surface Plasmon Resonance (TFBG-SPR) sensor, which has been specifically engineered for simplicity, reliability, and versatility—qualities that render it particularly suitable for environmental and biomedical applications [38]. In contrast to conventional FBG sensors, which primarily measure mechanical or thermal variations, the TFBG-SPR sensor leverages Surface Plasmon Resonance through light–plasmon interactions in a metallic coating. This design substantially enhances sensitivity and facilitates high-precision measurements in liquid media [146]. A key advantage of TFBG-SPR technology is its streamlined optical configuration, which eliminates the need for a Kretschmann prism—traditionally required in classical SPR systems. The addition of a gold coating on the fiber grating enhances compactness and facilitates integration with diverse optical platforms. Recent advances, including automated cladding mode selection algorithms, have reduced operator-induced errors and simplified calibration procedures [147]. Experimental studies have demonstrated the high sensitivity of TFBG-SPR sensors, with reported values reaching −6887 dB/RIU and a detection resolution down to 3 × 10^−6^ RIU [148]. These characteristics highlight their potential for real-time, high-precision chemical sensing.

Although TFBG-SPR sensors have been applied in various biomedical and environmental fields—such as cancer diagnostics, hormone monitoring, and neurotransmitter detection [149,150,151]—this review focuses specifically on their utility in pH measurement. In this context, the integration of SPR with pH-sensitive coatings enables robust and compact sensor designs capable of delivering accurate results under complex environmental conditions.

#### 3.3.1. Materials and Design Features

SPR sensors exploit the thin films of noble metals, such as gold and platinum, due to their exceptional plasmonic properties. Typically, these films—ranging from 30 to 50 nm in thickness—are crucial for achieving high sensitivity [38]. To further enhance pH detection, additional functional coatings, including polyaniline (PANI) and polyacrylic acid (PAA), are applied. These coatings operate via reversible proton exchange mechanisms, which modulate their optical and electrical properties in response to pH variations, thereby improving selectivity [39]. Moreover, advanced configurations—such as Tilted Fiber Bragg Grating systems [152] and measurement setups combining multimode fiber (MMF) with no-core fiber (NCF) (MMF-NCF-MMF systems) [153]—significantly enhance sensitivity and expand the applicability of SPR-based pH sensors across diverse fields [38].

#### 3.3.2. Measurement Range

**Figure 3:**
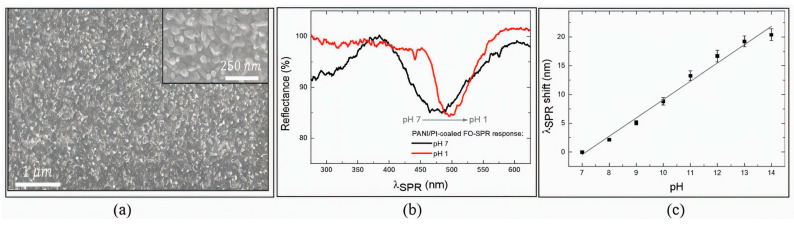
(**a**) SEM image of the uniform PANI coating on the fiber surface. (**b**) SPR spectra at pH 1 and 7 showing resonance shift due to pH response. (**c**) Linear correlation between SPR shift and pH (7–14), indicating high sensitivity in alkaline range [154].

SPR-based sensors facilitate pH measurements over a range from 2 to 12, demonstrating considerable versatility for diverse applications [38]. These sensors exhibit high sensitivity, as evidenced by the linear resonance wavelength shifts in response to pH variations, which ensures precise and reliable measurements. A representative example is shown in Figure 3, where a fiber-optic SPR sensor coated with polyaniline (PANI) demonstrates strong pH responsiveness [154]. The SEM figure (Figure 3a) confirms the uniform and continuous morphology of the PANI layer on the sensor surface. Reflectance spectra recorded at pH 1 and 7 (Figure 3b) show a clear shift in the SPR dip, indicating a strong modulation of the optical response due to protonation effects. The calibration curve (Figure 3c) reveals the near-linear dependence of the resonance wavelength shift on pH in the range of 7 to 14, confirming the sensor’s applicability for quantitative alkaline pH sensing.

#### 3.3.3. Sensitivity

The sensitivity is further enhanced in TFBG-SPR sensors that integrate tilted Fiber Bragg Gratings with SPR technology, thereby optimizing measurement accuracy [38]. In addition to their high sensitivity, SPR sensors exhibit operational stability under varying environmental conditions, maintaining consistent performance within a temperature range of 26 to 40 °C. This robustness renders them well suited for industrial and scientific applications [39]. Nevertheless, even with their intrinsic stability, it is advisable to incorporate temperature drift compensation mechanisms, especially for high-precision measurements, as minor temperature fluctuations can cause resonance wavelength shifts that compromise measurement accuracy.

To mitigate temperature drift effects, several strategies can be implemented, such as employing thermally stable materials, integrating additional temperature sensors for real-time correction, and applying specialized signal processing algorithms for data compensation [155,156].

#### 3.3.4. Advantages, Challenges, and Recent Developments

SPR sensors provide several distinct advantages that contribute to their effectiveness in diverse applications. Their label-free and real-time detection capabilities eliminate the need for external markers, enabling non-invasive monitoring with high precision [144].

SPR-based optical fiber pH sensors typically operate within a pH range of 2–12 (extendable to 14 in some configurations), offer a resolution of approximately 0.01 pH units, and demonstrate response times ranging from a few seconds to several minutes, depending on the system design and surface functionalization [157].

Their inherent sensitivity—enhanced by advanced configurations—permits the detection of subtle pH changes, with resolutions as fine as 0.01 pH units [157], while also supporting the multiplexed detection of other parameters, such as glucose, DNA, and proteins [158,159].

In terms of adaptability, SPR platforms can be configured for biomedical, chemical, and environmental monitoring, making them versatile tools for high-resolution sensing [45]. However, challenges remain, including the complexity of fabricating metal-coated structures, vulnerability to environmental fluctuations, and the reliance on costly noble metals like gold [44].

To overcome these challenges, recent developments have introduced innovative materials and structural strategies. Nanostructured enhancements—such as gold nanoparticles (AuNPs) and ultrathin functional films—have significantly improved detection sensitivity and lowered the limits of detection [160,161]. Layer-by-layer self-assembly methods using polyacrylic acid (PAA) and chitosan nanofilms have yielded multilayer systems with high stability and pH responsiveness, capable of detecting analytes like glyphosate at 0.05 μg/L and pH shifts of as small as 0.01 units [160,162].

**Figure 4:**
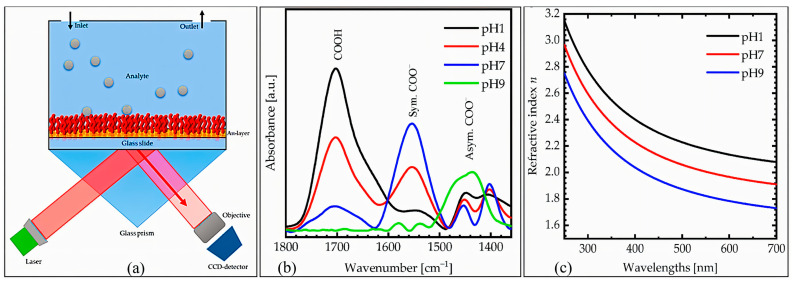
(**a**) Schematic of an SPR sensor in Kretschmann configuration with a PAA-coated Au layer. The red arrows indicate the laser path. The red layer shows the PAA coating, the blue area represents the analyte, and the purple region is the glass prism. Green and blue blocks indicate the laser source and CCD detector, respectively. (**b**) FTIR spectra of PAA brushes showing COOH/COO^−^ transitions at different pH levels. (**c**) Refractive index dispersion of PAA film as a function of wavelength and pH [163].

Additionally, Surface Plasmon Resonance Imaging (SPRI) has expanded the spatial resolution and sensitivity of SPR systems. As shown in Figure 4, PAA-modified gold layers exhibit reversible optical transitions in response to pH-dependent protonation, confirmed by FTIR spectra and dispersion curve shifts [163]. These properties establish PAA as an effective pH-sensitive interface, enabling reliable real-time monitoring in complex environments.

### 3.4. Interferometric Fiber Sensors

Interferometric optical fiber sensors function by detecting phase shifts in light resulting from refractive index variations along the optical path. For pH sensing, interferometers such as Mach–Zehnder or Fabry–Pérot are used, in which one arm or cavity is exposed to a pH-sensitive medium. Protonation changes alter the optical path length due to changes in the refractive index or physical expansion, creating interference fringes that shift with pH. These sensors are valued for their ultra-high resolution and ability to detect small variations in pH, especially when configured with narrow linewidth lasers or white light sources.

Interferometric fiber sensors have garnered significant attention due to their exceptional precision in measuring a variety of physical and chemical parameters [164]. These devices employ configurations such as Fabry–Perot, Mach–Zehnder, Michelson, and Sagnac Interferometers [165], with design and optimization focusing on factors such as feedback mechanisms, reflection schemes, and sensitivity [166]. Among these configurations, extrinsic Fabry–Perot interferometry provides superior sensitivity—particularly in pressure measurement applications [167]. Recent advancements have focused on enhancing the figure of merit, defined as the balance between sensitivity and fringe width, thereby improving the detection of minute changes in the refractive index. This capability renders them highly suitable for applications including gas pressure monitoring, dilute solution analysis, and pH concentration measurement [165].

Furthermore, these sensors are widely recognized for their real-time detection capabilities in pH sensing due to their high sensitivity and compact form factor. As interferometric techniques continue to evolve, they remain accurate and versatile tools for a wide range of sensing applications, offering rapid and precise measurements.

#### 3.4.1. Materials and Design Features

Intrinsic Fabry–Perot Interferometers (IFPIs) are fabricated by inscribing microcavities along the core of optical fibers using femtosecond laser pulses. When operated at optimal power levels (up to 60 mW), this process yields high-quality cavities with excellent spectral visibility [168]. These cavities are subsequently coated with a palladium-doped titanium dioxide (Pd-TiO_2_) thin film, where palladium nanoparticles act as catalysts for the hydrogen-to-hydride conversion, generating measurable strain that correlates with pH variations. To ensure a uniform and stable coating, the dip coating technique is combined with a sol–gel process, further enhanced by the addition of Pluronic^®^ F-127 (Sigma-Aldrich, St. Louis, MO, USA) copolymer, which improves homogeneity and reduces the number of defects [42]. The resulting sensor structure features a dense and uniform thin film layer, as verified by SEM imaging, and experimental results demonstrate a fast, reversible response (~7 s) across a pH range of 1.0 to 7.0 [164]. These characteristics make the IFPI design a robust and efficient platform for pH sensing.

**Figure 5:**
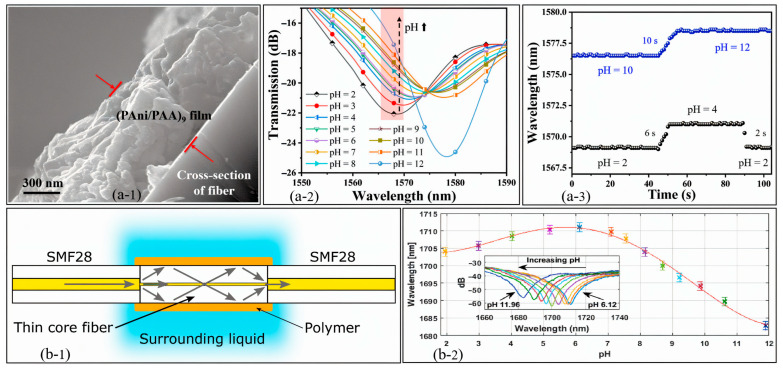
(**a-1**) SEM image showing the porous surface of the PAni/PAA coating (~1 μm), enabling effective pH interaction. (**a-2**) Transmission spectra under pH 2–12, showing a clear redshift with increasing pH: pink area denotes analyzed region. (**a-3**) Fast and reversible spectral response during pH cycling, confirming real-time performance [153]. (**b-1**) Working principle of the Mach–Zehnder interferometric (MZI) sensor based on a thin-core fiber spliced between two SMF28 fibers and coated with a pH-sensitive hydrogel. Gray arrows denote different modes of propagating radiation. (**b-2**) Spectral response of the MZI sensor showing wavelength shifts correlated with pH changes across a wide dynamic range (1.95–11.89) [41].

Recent advancements introduced a fast-response, wide-range optical pH sensor based on a no-core fiber (NCF) spliced between two single-mode fibers (SMFs) in an SNS configuration [153]. A pH-sensitive PAni/PAA multilayer film is applied using layer-by-layer (LBL) self-assembly, forming a ~1 μm porous coating clearly visible in Figure 5(a-1), which facilitates strong interaction with the surrounding medium.

The optical response of the sensor demonstrates a clear redshift in the transmission spectrum with increasing pH levels, as shown in Figure 5(a-2), indicating high pH sensitivity across a wide detection range. Additionally, Figure 5(a-3), presents the dynamic performance, confirming the sensor’s rapid and reversible response when cycling between acidic and basic conditions. These results highlight the sensor’s strong sensitivity and fast response time for pH monitoring with minimal thermal interference [153].

Mach–Zehnder interferometric (MZI) fiber sensors, particularly those incorporating thin-core fiber segments, offer a highly effective platform for pH detection. These sensors are fabricated by splicing a short section of thin-core fiber between two standard single-mode fibers (SMF28), followed by the application of a pH-responsive hydrogel coating. This biocompatible hydrogel expands or contracts in response to pH-induced ionic changes, leading to measurable variations in the local refractive index and resulting in spectral shifts in the interference pattern.

As illustrated in Figure 5(b-1,b-2), the sensor design allows for effective interaction between the evanescent field and the surrounding medium through the hydrogel layer, enabling high sensitivity to pH fluctuations. Experimental results show a clear and repeatable spectral shift across a wide pH range (1.95–11.89), confirming the sensor’s broadband and reversible response capabilities [41]. The combination of the compact form factor, responsiveness to environmental changes, and compatibility with biological systems makes MZI-based sensors well suited for both biomedical and environmental applications.

Multicore fibers (MCFs) integrate multiple optical cores within a single cladding, offering considerable structural and functional versatility [169,170]. These interferometric sensors provide significant advantages, such as enhanced integration capabilities and spatial multiplexing, which enable the simultaneous measurement of multiple parameters.

MCF-based sensors are specifically designed to monitor various physical and chemical parameters, including pH, by analyzing differential optical responses across individual cores [171,172,173]. Their compact design, coupled with the ability to measure multiple properties simultaneously, renders them a highly versatile and efficient solution for sensing applications.

#### 3.4.2. Measurement Range and Sensitivity

The performance of interferometric fiber pH sensors is contingent upon their design and the materials utilized. Intrinsic Fabry–Perot Interferometers exhibit high sensitivity, largely attributable to the catalytic activity of Pd nanoparticles, which facilitates precise pH measurements. These sensors are particularly effective in environmental and biomedical applications that require rapid response times [105,174,175].

The responsiveness of the hydrogel in thin-core Mach-Zehnder Interferometers enables a broad measurement range from 1.95 to 11.89, with reported sensitivities of approximately 11 nm per pH unit in specific intervals. Consequently, these sensors are well suited for applications that require both an extensive pH detection range and high sensitivity [41].

The differential response among the cores in multicore fiber interferometers significantly enhances sensitivity and facilitates multiplexed pH measurements, although precise sensitivity values depend on the specific configuration and materials employed [173,176,177,178].

#### 3.4.3. Advantages, Challenges, and Recent Developments

Interferometric fiber sensors demonstrate exceptional sensitivity and precision, detecting minute pH variations across a wide range (1.95 to 11.89) with a resolution of up to 10^−5^ RIU. Their rapid response times—ranging from 1.58 to 15.77 s—and typical sensitivity of approximately 11 nm per pH unit, render them highly suitable for critical applications such as biomedical diagnostics and environmental monitoring [147,148].

Interferometric pH sensors typically operate across a wide range of 1.95 to 11.89, achieving sensitivities of around 11 nm per pH unit and response times of between 1.6 and 15.7 s, depending on the interferometric design and sensing layer properties [41].

The compact size and mechanical flexibility of these sensors enable seamless integration into in vivo and embedded systems, thus broadening their applicability for real-time, minimally invasive pH detection across diverse conditions [179,180,181].

Despite their benefits, several challenges must be addressed to ensure consistent long-term operation. One primary issue is the durability of the sensing layer, particularly in harsh or biologically active environments. Factors such as temperature fluctuations, ionic strength variation, and biofouling can degrade sensor performance over time [182,183]. Ongoing research efforts focus on developing protective coatings and robust structures to mitigate these influences and improve operational lifespan [184,185].

In parallel, recent developments in interferometric fiber sensor technology have led to notable improvements in performance, scalability, and manufacturing efficiency. Material innovations, such as the incorporation of biocompatible and responsive hydrogel layers, as well as nanomaterials like palladium-doped titanium dioxide (Pd-TiO_2_), have significantly enhanced pH responsiveness and long-term stability in biomedical applications [178,186].

Structural enhancements, particularly the use of multicore fiber designs, have enabled multifunctional sensors capable of simultaneously monitoring pH and other parameters (e.g., temperature), increasing the versatility of these systems in complex environments [187].

Advanced fabrication techniques, including femtosecond laser direct writing, have further improved sensor reproducibility and precision, while supporting scalable, cost-effective manufacturing. These innovations address one of the remaining barriers to widespread adoption: the complexity and cost of production [188,189]. Together, these advancements position interferometric fiber sensors as robust and adaptable platforms for next-generation pH sensing in demanding industrial, biomedical, and environmental applications.

### 3.5. FBG-Based pH Sensing Technologies

FBG-based pH sensors incorporate Bragg Gratings inscribed in the fiber core, which reflect specific wavelengths based on the periodic modulation of the refractive index. When coated with a pH-sensitive material, the swelling or contraction of the coating—due to proton exchange—induces strain or refractive index changes in the grating region, resulting in a measurable shift in the reflected Bragg wavelength. Though inherently sensitive to temperature and strain, these sensors can be designed for pH selectivity through functional material engineering, making them suitable for harsh and high-precision environments.

Fiber Bragg Grating sensors have emerged as a transformative technology across a wide range of sensing applications [190]. Widely utilized in biomedical monitoring, structural health assessments, and ultrasonic wave detection [191], their performance in pH sensing is especially notable. FBG sensors deliver high sensitivity and maintain reliable operation in diverse and challenging environments. These sensors detect shifts in the Bragg wavelength (λB) that result from pH-induced changes in the surrounding material, offering precise and responsive monitoring of environmental conditions. Their ability to operate in real time under varying chemical and physical influences makes them particularly valuable for pH sensing applications.

The performance of FBG-based pH sensors is contingent upon several key factors, including the measurable pH range, the sensitivity of the materials employed, and the overall design architecture. Some sensors are optimized for acidic environments, typically operating within a pH range of 3 to 7, while others are engineered for broader applications, extending the operational range from 2 to 12 [192,193].

Sensitivity levels range widely—from approximately 12.16 pm/pH to 117 pm/pH—depending on the material composition and structural design [192,194]. A diverse array of stimuli-responsive materials has been employed in FBG sensor designs, including hydrogels (e.g., PVA/PAA and PMMA) and polymers (such as PDDA/PAA and polyaniline [PAni]). These materials swell in response to pH changes, inducing strain in the grating and thereby enhancing measurement resolution [194].

Beyond synthetic polymers, acid–base indicators such as Bromothymol Blue (BTB) are being investigated for incorporation into FBG-based sensors. BTB exhibits distinct colorimetric changes in response to varying pH levels and has been successfully applied in polymeric and optical sensing systems; however, its integration within FBG configurations remains an emerging area of research with significant potential for future development [195,196].

Certain FBG-based sensors demonstrate rapid response times, with some achieving rise times as brief as 10 s. However, due to the inherent temperature cross-sensitivity of optical fibers, many sensor designs integrate temperature compensation mechanisms to preserve measurement accuracy under fluctuating thermal conditions.

Table 3 presents a concise summary of various FBG-based pH sensing techniques, outlining their operating principles and primary advantages.

These sensors utilize hydrogel coatings, polymeric materials, and functional films to improve sensitivity and achieve rapid response times. Certain designs are optimized for a broad pH detection range, whereas others emphasize enhanced stability and repeatability for long-term operation. Table 4 below summarizes a comparative analysis of various FBG-based pH sensors, highlighting key differences in their properties and applications.

FBG-based pH sensors provide a versatile and reliable method for pH measurement, with significant potential for enhancement through innovations in materials and sensor design optimization. Future developments could extend the measurable pH range, refine temperature compensation techniques, and integrate these sensors into multi-parameter monitoring systems, thereby further improving their performance across a diverse array of applications.

#### 3.5.1. Materials and Design Features

**Figure 6:**
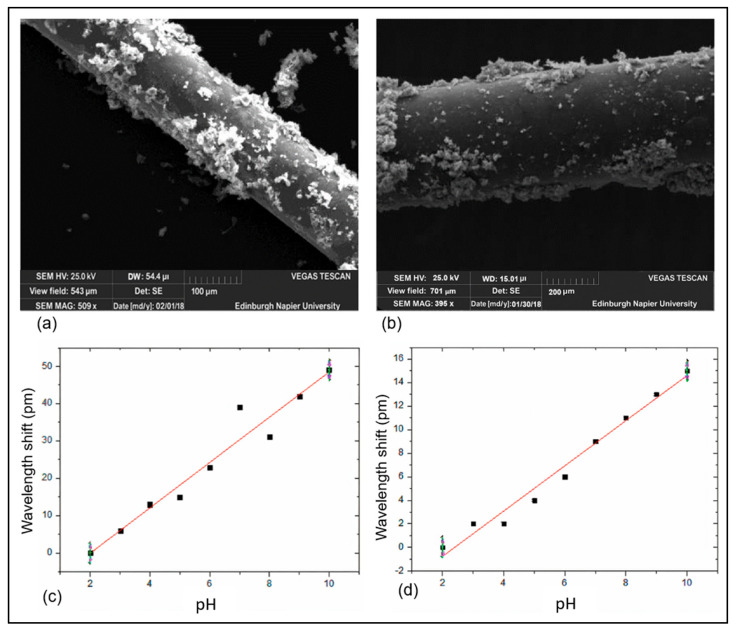
(**a**) SEM of FBG with evaporated GO: uneven coating and weak adhesion. (**b**) SEM of FBG with co-electroplated GO: more uniform and strongly bonded layer. (**c**) Calibration curve for evaporated GO-coated FBG: higher sensitivity (6.1 ± 0.5 pm/pH). (**d**) Calibration curve for co-electroplated GO-coated FBG: lower sensitivity (1.9 ± 0.1 pm/pH) with better stability [208].

FBG sensors for pH measurement utilize coatings that expand or contract in response to pH variations, thereby inducing strain on the grating and shifting its reflected wavelength. Frequently employed materials include hydrogels (e.g., PVA/PAA, PEG, and sodium alginate) [192,204], conductive polymers (such as polyaniline and GO/PVA composites) [193,203], and nanostructured films (including graphene oxide, Cu/WS_2_, and PDMS layers) [205,206]. The selection of the coating material significantly influences the sensor’s sensitivity, stability, and response time. Moreover, design enhancements such as the implementation of microfiber Bragg Gratings (μFBGs) [207] and capillary-assisted configurations [200] further improve performance in specialized applications. To illustrate the influence of the coating morphology and deposition method, Figure 6 presents a comparative analysis of FBG sensors coated with graphene oxide (GO) and hydrogel using two techniques: evaporation and co-electroplating [208]. SEM micrographs (Figure 6a,b) show distinct surface textures—evaporation results in a loosely attached, non-uniform layer, while co-electroplating produces a more compact and adherent coating.

These morphological differences directly affect sensor performance. As shown in the calibration curves (Figure 6c,d), the evaporated GO yields a higher pH sensitivity of 6.1 ± 0.5 pm/pH, whereas the co-electroplated GO provides better mechanical stability, with a sensitivity of 1.9 ± 0.1 pm/pH [208]. These findings underscore the critical role of surface functionalization and material integration in optimizing FBG-based pH sensors for biomedical and environmental applications.

#### 3.5.2. Measurement Range and Sensitivity

FBG-based pH sensors can be engineered to cover a broad pH range, contingent upon the choice of coating material and interrogation technique. For example, hydrogel-coated FBG sensors typically operate within the acidic range (pH 3–7) and offer a sensitivity of 12.16 pm/pH [192]. In contrast, polyaniline-coated FBG sensors extend the measurement range to pH 2–12 while providing rapid response and high stability [193]. Additionally, graphene oxide/PVA hybrid coatings can enhance sensitivity up to 0.69 nm/pH and achieve response times of less than 10 s [207]. Microfiber Bragg Gratings (μFBGs) employing sodium alginate demonstrate a high sensitivity of 62.8 pm/pH along with a detection resolution of 0.096 pH, rendering them particularly suitable for microfluidic applications [206]. Finally, super-porous polymer coatings enable reversible pH sensing in extreme environments within a range of pH 3–8 [209].

#### 3.5.3. Advantages, Challenges, and Recent Developments

FBG-based sensors provide versatile advantages that enable their effective deployment across a wide range of pH sensing applications. Their compactness and remote sensing capabilities allow seamless integration into bioreactors, environmental monitoring systems, and industrial pipelines [210]. Enhanced sensitivity can be achieved using microfiber configurations, enabling high-resolution and real-time performance [206]. Furthermore, FBG sensors enable dual-parameter detection, particularly of pH and temperature, which helps to reduce the signal interference due to thermal effects [205,208]. Their biocompatibility, demonstrated through materials such as polyethylene glycol (PEG), polyaniline, and sodium alginate, makes them suitable for in vivo applications [44,199].

Typically, FBG-based pH sensors operate within a pH range of 2–12, exhibit sensitivities from 12 to 117 pm/pH, and demonstrate response times ranging between 10 and 30 s, depending on the coating type and grating configuration [192,193].

However, several challenges remain. Uncompensated FBG sensors are prone to temperature cross-sensitivity, which necessitates correction mechanisms or dual-sensing approaches [211]. Some hydrogel coatings exhibit slow response times due to prolonged equilibration, limiting their usefulness in real-time monitoring [199]. In addition, material degradation and insufficient coating integrity can compromise long-term stability in harsh environments [193,210].

To overcome these limitations, recent research has advanced FBG technology through hybrid configurations, nanostructured coatings, and miniaturization techniques. Dual-function sensors that simultaneously monitor pH and temperature have shown improved thermal stability. For example, PDMS-coated FBGs demonstrated a 34% increase in temperature sensitivity, significantly mitigating thermal interference and enhancing accuracy in dynamic environments [205].

Hydrovoltaic FBG sensors represent another innovation, combining FBG elements with microcapillaries to detect subtle pH shifts in micro-scale aqueous samples (<2 μL), offering a compelling solution for biochemical sensing where sample volume is limited [200]. Similarly, photonic FBG sensors utilizing pH-sensitive chromophores rely on photothermal effects, where pH-induced temperature changes shift the Bragg wavelength, allowing indirect optical pH detection [212].

Miniaturization efforts have also led to ultra-compact FBG sensors fabricated via electrostatic self-assembly, achieving sensitivities of −72 pm/pH in fundamental modes and up to −265 pm/pH in higher-order modes. These developments highlight the feasibility of FBG-based systems for intracellular and cellular-level pH monitoring [44].

### 3.6. Luminescence Lifetime-Based Sensors

Fluorescence lifetime-based sensors determine pH by measuring the time for which a fluorophore remains in the excited state before emitting a photon. This lifetime is influenced by pH-sensitive environmental factors such as the proton concentration, which alters non-radiative decay rates. Unlike intensity-based systems, lifetime measurements are less affected by optical path loss or dye concentration, making them more robust in complex or turbid media. Optical fibers serve as conduits for excitation and emission, enabling remote deployment in miniaturized sensing platforms.

The development of luminescence lifetime-based pH sensors has enabled highly sensitive and reliable measurements, particularly in challenging environments such as biological tissues and turbid solutions. By eliminating the dependence on signal intensity, these sensors offer improved reproducibility, stability, and long-term performance compared to traditional fluorescence sensors. Table 5 compares various pH sensing methods based on luminescence lifetime techniques, demonstrating their precision, stability, and minimal interference relative to conventional intensity-based sensors.

Advances in signal interrogation, such as the use of Empirical Mode Decomposition (EMD) and digital filtering in OFDR systems, have notably enhanced the resolution and efficiency of distributed fiber-optic sensors. These methods may support future real-time pH sensing in compact and cost-effective formats [216].

#### 3.6.1. Materials and Design Features

Luminescence lifetime-based pH sensors rely on optically responsive materials—primarily metal complexes, nanoclusters, and polymeric matrices—that exhibit stable and tunable luminescent properties under varying environmental conditions. Among these, ruthenium(II) and iridium(III) polypyridyl complexes have been extensively studied for their strong phosphorescence, thermal and chemical stability, and customizable emission lifetimes, achieved through ligand modification [213]. A recent innovation in this field is the [Ru(DCB)_2_DEAMB] complex, which incorporates carboxylate and tertiary amine groups to provide a broad dynamic pH response range (3.5–8.5). The emission lifetime shifts from 335 ns at pH 3.5 to 429 ns at pH 8.5, demonstrating high sensitivity and reversibility under aqueous conditions. To enhance robustness and reusability, the complex is covalently immobilized onto TentaGel^®^ M Br (Rapp Polymere GmbH, Tübingen, Germany) polymer beads through a nucleophilic substitution reaction followed by a Hofmann elimination step that restores the proton-sensitive tertiary amine functionality [213]. Such a configuration has demonstrated repeatable and precise pH detection based on luminescence lifetime and is fully compatible with phase-sensitive optical fiber interrogation systems. The sensor has been successfully validated under bioreactor conditions simulating microbial fermentation, where the phase shift signal recorded by the optical sensor closely correlated with the pH variations detected by a standard electrode, confirming its accuracy and stability. A well-defined calibration curve further supports its reliability across the operational pH range [213]. To further improve stability and biocompatibility, these metal complexes are often embedded within PEG-based copolymers or silica matrices (SiO_2_), which enhance their mechanical performance and suitability for biomedical and environmental applications [218]. Additionally, platinum nanoclusters (Pt NCs) have shown promise due to their small size and excellent photostability, offering high-resolution pH detection within physiological ranges [219]. Other systems incorporate dual-emission Ir(III) complexes into hydrophilic polymers, allowing for self-referencing intracellular sensing with high spatial and temporal resolution [41]. The adoption of ratiometric sensor architectures, using two independent luminescent emissions, improves measurement accuracy by correcting for fluctuations in excitation intensity and environmental interference [40]. Moreover, the increasing integration of these sensors with optical fiber platforms enables real-time, in situ pH monitoring in biological tissues, living cells, fluids, and harsh environments [220].

#### 3.6.2. Measurement Range and Sensitivity

A critical feature of pH sensors is their ability to accurately detect changes across relevant pH ranges with high sensitivity, a capability that varies depending on the sensing material and its interaction with protons. For example, wide-range sensors such as ruthenium(II) polypyridyl-based systems reliably detect pH changes across a broad spectrum (pH 3–10), making them suitable for both environmental and biomedical applications [213]. In contrast, platinum nanocluster-based sensors offer high precision within the physiological pH range (6.02–7.54), rendering them ideal for medical diagnostics and intracellular studies [221]. Advanced polymeric probes have been developed for subcellular sensing to monitor pH fluctuations in organelles such as lysosomes and mitochondria within the pH range of 4.5–7.5 [47]. Furthermore, high-resolution systems employing techniques like time-resolved fluorescence spectroscopy and frequency–domain luminescence imaging achieve a pH resolution of 0.01–0.05 units, which is essential for detecting subtle changes in dynamic biological systems [222]. Additionally, these sensors typically demonstrate rapid response times—ranging from a few seconds to 2–3 min—depending on the diffusion rate of hydrogen ions into the sensing matrix [46]. These capabilities make luminescence lifetime-based pH sensors particularly valuable for precise, real-time measurements in complex environments.

#### 3.6.3. Advantages, Challenges, and Recent Developments

Luminescence lifetime-based pH sensors offer distinct advantages over traditional intensity-based approaches. Unlike intensity measurements, lifetime responses are largely independent of dye concentration, excitation fluctuations, and photobleaching, making them highly stable and reproducible [221].

Luminescence lifetime-based pH sensors typically operate within a pH range of 3 to 10, offer sensitivities between 0.01 and 0.05 pH units, and demonstrate response times ranging from a few seconds up to 3 min, depending on the phosphorescent material and detection system [217].

These systems exhibit excellent sensitivity and photostability, which supports their long-term deployment in complex environments [46]. The use of fiber-optic and imaging-based platforms further enables continuous, remote, and in situ monitoring, while recent innovations in multiplexing allow for the simultaneous detection of multiple analytes—such as pH, O_2_, and CO_2_—broadening their utility in biomedical and environmental contexts [223,224].

Nonetheless, these sensors face key challenges. Fabrication and calibration are often complex due to the sophisticated nature of phosphorescent dyes and the need for precise instrumentation. Many systems require the integration of self-calibrating components and advanced imaging technologies that can be costly and technically demanding [47,225]. Additionally, some lifetime-based sensors operate within limited pH ranges—for example, platinum nanoclusters from pH 6.02 to 7.54 [221] or polymeric probes from 4.5 to 7.5 [47]—which may constrain their use in broader applications. Environmental interferences such as oxygen quenching, temperature variability, and matrix effects can also compromise accuracy and demand robust sensor design refinements [226].

Recent advancements have addressed many of these limitations through innovations in materials, system design, and detection methodology. Nanostructured sensing materials such as rare earth-doped nanoparticles and metal–organic frameworks (MOFs) have significantly improved photostability and extended emission lifetimes [227]. Dual-emission sensors incorporating iridium-based phosphorescent channels and fluorescein isothiocyanate (FITC) enable self-referencing and accurate intracellular pH mapping, even under dynamic biological conditions [47]. These have been successfully applied in live zebrafish models and intracellular systems, demonstrating both high spatial and temporal resolution.

From a systems perspective, the transition from time–domain imaging to more affordable frequency–domain techniques has enabled real-time monitoring using consumer-grade cameras and portable platforms [225]. These developments have made lifetime-based sensors more accessible and scalable. Additionally, designs that allow for the co-detection of pH and oxygen further enrich the contextual analysis of biological and environmental samples [223]

Together, these innovations represent significant progress in making luminescence lifetime-based pH sensors more stable, selective, and suitable for real-time, in vivo, and field applications.

### 3.7. Optical Fiber Evanescent Wave Sensors

Evanescent wave sensors exploit the exponentially decaying light field that extends beyond the core of an optical fiber under total internal reflection conditions. When the cladding is removed or modified, and a pH-sensitive material is deposited, the evanescent field interacts directly with the analyte. Changes in pH modify the absorption or refractive index of the sensing layer, leading to detectable attenuation or spectral shifts. These sensors allow for highly localized and rapid pH detection, particularly useful in microfluidic and biomedical systems.

Recent advancements in OFEWSs have expanded their utility across diverse sensing domains. While pH monitoring remains a primary application, these sensors have also demonstrated effectiveness in detecting gases and chemicals—such as ethanol and organic compounds for industrial process control and fuel quality assessment [228]—as well as in detecting toxic metals such as mercury to enhance environmental safety [229]. Comparative analyses, as summarized in Table 6, highlight key research areas, including temperature and humidity sensing, light propagation, chemical detection, and innovative improvements in fiber-optic sensor configurations, with each study exploring distinct approaches to enhance the accuracy of pH detection using evanescent wave-based optical fiber technology.

Evanescent wave-based optical fiber sensors are pivotal in ion and biosensing, enabling the detection of potassium ions for agricultural and biochemical applications [232] and facilitating the real-time monitoring of biomolecules and DNA in medical diagnostics [233]. In addition, ZnO-coated fibers enhance temperature and humidity measurements [234], while polymer optical fibers (POFs) offer superior durability in harsh industrial environments [235]. Furthermore, photonic bandgap (PBG) sensors improve biosensing selectivity, thereby expanding the potential for precise bioanalytical applications [236].

Modified fiber surfaces facilitate analyte detection through the monitoring of light intensity variations, for example, graphene-enhanced polymers improve glucose sensitivity [237], polyaniline nanofibers are effective in detecting β-lactam antibiotics [238], and nanocrystalline aluminum oxide coatings significantly increase sensitivity to volatile organic compounds (VOCs), chemical substances that readily evaporate at room temperature [239], while gold nanoparticles further enhance the detection of potassium ions in agricultural applications [232].

Recent advancements in optical fiber pH sensors have leveraged evanescent wave absorption to achieve precise aqueous pH measurements [21]. Moreover, the integration of a CRISPR-Cas13a biosensor with a hybridization chain reaction (HCR) has enabled the ultra-sensitive detection of SARS-CoV-2 at levels as low as approximately 6 copies/μL within one hour—thereby underscoring its potential for point-of-care diagnostics [240]. Additionally, the development of reusable optical fibers, which maintain performance over more than 100 cycles with minimal signal loss, further demonstrates the practical viability and robustness of these technologies.

Accurate, real-time pH monitoring is essential for environmental, medical, and industrial applications [241], yet traditional electrochemical sensors often suffer from drift and a limited lifespan [241]. In contrast, OFEWSs leverage evanescent fields to deliver high sensitivity, enable real-time monitoring, and facilitate miniaturization for precise pH detection [85].

#### 3.7.1. Materials and Design Features

**Figure 7:**
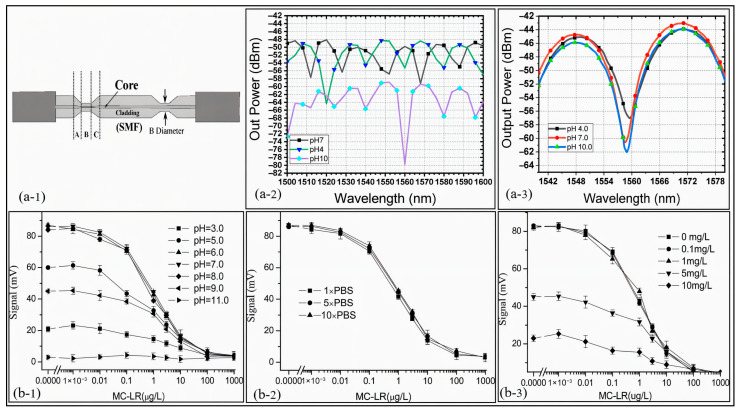
Optical fiber sensor designs and pH response (**a-1**): Schematic of a tapered Mach–Zehnder Interferometer (MZI) fiber structure with regions A, B, and C indicating different tapered zones and cladding/core transitions for enhanced evanescent field interaction. (**a-2**): Transmission spectra at pH 4 (purple), 7 (green), and 10 (blue). (**a-3**): Resonance wavelength shift at 1559 nm for pH 4.0 (black), 7.0 (red), and 10.0 (orange). [242]. (**b-1**): Signal vs. MC-LR concentrations under pH 3–11, indicated by different symbols. (**b-2**): Signal response for 1×, 5×, and 10× PBS dilutions. (**b-3**): Signal variation with MC-LR concentrations from 0 to 10 mg/L. [243].

The performance of OFEWSs for pH sensing depends primarily on the fiber structure, cladding modifications, and the sensing materials incorporated into the design. Various optical fiber configurations have been developed to enhance evanescent wave interaction and improve pH sensitivity: tapered fibers reduce the core diameter to increase the evanescent field exposure [85], D-shaped fibers involve partial cladding removal to allow direct interaction with pH-sensitive coatings [230], microfiber-based sensors employ ultra-thin fibers to maximize the surface interaction and chemical sensitivity [234], and photonic bandgap (PBG) sensors utilize periodic dielectric structures to control and enhance evanescent wave propagation, thereby improving measurement accuracy [236]. Equally importantly, the coating materials play a crucial role in determining the sensor’s response and stability, with options including polyaniline (PANI), a conductive polymer that undergoes reversible color and refractive index changes in response to pH variations [230]; sol–gel coatings, which immobilize pH-sensitive dyes to ensure long-term stability [230]; fluorescent pH indicators, which exhibit high selectivity through shifts in their emission spectra [233]; zinc oxide (ZnO) nanorods, which enhance both optical and chemical sensitivity and are ideal for pH and temperature sensing [234]; and advanced nanocomposite and polymer layers, such as graphene-based coatings, which offer improved durability and sensitivity [85]. Figure 7(a-1–a-3) present the structure and optical response of a tapered Mach–Zehnder interferometric fiber sensor [242]. The central waist (a-1) increases the evanescent field penetration, while spectral plots (a-2, a-3) reveal wavelength shifts under different pH conditions, demonstrating strong refractive index sensitivity.

Figure 7(b-1–b-3) highlight the behavior of evanescent wave immunoassay sensors under varying pH and analyte concentrations [243]. The signal intensity decreases with increasing analyte levels, and optimal sensitivity is observed in the physiological pH range (6–8), confirming the significance of the fiber surface chemistry in pH detection.

#### 3.7.2. Measurement Range and Sensitivity

Optical fiber evanescent wave sensors can be engineered to operate over a wide range of pH values, contingent upon the selected fiber configuration and sensing materials. Various materials and fiber structures contribute to differing detection ranges; for instance, PANI-coated fibers reliably detect pH values of between 2 and 10 with a stable response and excellent reversibility [230], fluorescence-based sensors extend this range from pH 3 to 11 while maintaining high selectivity [233], and metal oxide as well as sol–gel sensors offer tunable ranges—often spanning pH 2 to 12—depending on their specific material composition [230,234]. Furthermore, to enhance measurement accuracy, several strategies have been developed to improve sensitivity, including the use of tapered fiber geometries that increase light penetration into the sensing material, nanomaterial coatings such as ZnO nanostructures and graphene-based films that amplify evanescent field interactions, and the integration of Surface Plasmon Resonance techniques, which notably boost response sensitivity, particularly in biomedical pH sensing applications [85,233,236].

#### 3.7.3. Advantages, Challenges, and Recent Developments

Optical fiber evanescent wave sensors (OFEWSs) offer distinct advantages over conventional pH sensing techniques due to their exceptional sensitivity to changes in the refractive index, wavelength, polarization, chemical composition, and temperature. This sensitivity allows for the accurate, real-time monitoring of chemical environments with rapid response times, driven by the strong interaction between the evanescent field and the analyte [85]. Their compact and flexible design facilitates integration into portable and lab-on-a-chip systems, while their non-invasive and remote sensing capabilities make them ideal for environmental and biomedical applications [230,244].

Recent innovations have further enhanced sensor performance through the application of nanomaterial coatings such as zinc oxide (ZnO), graphene, and polyaniline (PANI). These materials extend operational lifetimes (up to 40 days at 4 °C), improve long-term stability, and reduce measurement error from ±0.05 nm to ±0.02 nm [217,245]. Notably, graphene can increase sensitivity by approximately 50% via electronic noise suppression, achieving a sensitivity of up to −27.8 pm/ppm [246,247].

Evanescent wave-based pH sensors typically operate over a pH range of 2 to 12, with sensitivities that vary depending on the coating and fiber structure—for example, reaching −27.8 pm/ppm using graphene coatings—and offer rapid response times on the order of a few seconds [85,233,236].

However, challenges persist. The long-term stability of organic dye-based coatings remains limited due to photobleaching and chemical leaching [248]. Moreover, environmental factors—such as ionic strength variations, temperature fluctuations, and turbidity—can interfere with accuracy, especially in complex aquatic systems [85]. To mitigate these effects, recent efforts have focused on developing advanced error compensation algorithms, including IEEE 1451.2-based models, temperature coefficient adjustments, and reference fiber techniques to counteract signal instability [244].

Recent research has also introduced structural and functional advancements to improve reliability and measurement precision. Multi-layer polymer optical fibers with roughened interlayer surfaces have enhanced evanescent wave absorption, improving both accuracy and sensitivity [248]. The integration of photonic bandgap (PBG) structures into OFEWS designs provides superior control over light–matter interactions, further enhancing selectivity and performance [236].

Additional progress includes the embedding of metal-doped fluorescent nanocomposites—such as quantum dots—which shorten response times and maintain optical stability under dynamic sensing conditions [233]. Collectively, these advancements represent a major step forward in establishing OFEWSs as robust, selective, and adaptable tools for real-time pH monitoring in challenging environmental and biological settings.

### 3.8. Comparative Summary of Sensor Performance

To provide a unified comparison across all sensor types, Table 7 summarizes the key performance metrics—including the pH measurement range, sensitivity, and response time—for each optical fiber-based pH sensing approach discussed in this review.

### 3.9. Future Research Directions

Future research in optical fiber sensor technologies should prioritize advancements in optical and optoelectronic interrogation schemes, particularly through the development and optimization of Addressed Fiber Bragg Structures (AFBSs). AFBSs simplify the interrogation process by encoding sensor information into frequency-separated spectral components, thereby facilitating efficient multiplexing and significantly improving measurement accuracy [249,250]. Unlike traditional FBG interrogation methods, which rely on high-resolution optical spectrometers or expensive tunable lasers, AFBSs can be effectively interrogated using cost-efficient microwave photonic techniques, enhancing real-time sensing capabilities and considerably reducing the overall system deployment costs. This approach promises to enable the broader adoption of optical fiber sensor networks across various sectors [218,219].

The further exploration and development of phase-shifted AFBSs is expected to significantly enhance the sensitivity and resolution of interrogation systems, thereby offering considerable benefits for dynamic sensing applications such as vibration monitoring, temperature detection, and structural health assessment [220]. Moreover, integrating AFBSs with microwave photonic processing could substantially broaden their applicability, particularly in critical industrial domains where rapid and reliable sensing is essential [219,251].

Future research should actively investigate the integration of advanced functional materials—such as nanostructured films, graphene-based coatings, and hybrid polymers—to enhance sensor performance in terms of sensitivity, selectivity, and long-term stability. Incorporating artificial intelligence and machine learning into data processing and analysis is expected to improve measurement accuracy, enable predictive analytics, and facilitate proactive maintenance strategies in industrial environments, while concurrent efforts toward sensor miniaturization and the development of multi-parameter sensing systems capable of simultaneously measuring variables such as pH, temperature, and pressure could significantly broaden the versatility and application scope of optical fiber sensors.

From an economic standpoint, the adoption of AFBS technology offers a substantial advantage by significantly reducing the overall cost of fiber-optic sensor systems. Unlike conventional interrogation systems—which generally require expensive instrumentation and extensive computational resources—AFBS technology employs cost-effective microwave photonic methods for signal interrogation, thereby facilitating large-scale deployment and enhancing commercial viability. This positions optical fiber sensors as practical, economical solutions that can be readily applied across a broad spectrum of scientific and industrial contexts [218,219,252].

## 4. Conclusions

Optical fiber sensors have revolutionized pH measurement by offering highly sensitive, real-time, and non-invasive monitoring capabilities. This review examines major advancements across a range of optical fiber-based sensing techniques—including fluorescence-based, absorbance-based, evanescent wave, interferometric, Fiber Bragg Grating, Surface Plasmon Resonance, and luminescence lifetime-based sensors—each providing unique benefits such as high precision, exceptional sensitivity, and robust environmental adaptability that render them invaluable for biomedical diagnostics, environmental monitoring, and industrial applications.

Despite these advantages, challenges persist, including temperature cross-sensitivity, material stability, and fabrication complexity. Addressing these issues necessitates continued research into novel materials, advanced fabrication techniques, and integrated sensor systems incorporating artificial intelligence. Recent innovations, such as nanostructured coatings, multifunctional sensing platforms, and smartphone-based real-time monitoring, have further enhanced the practicality and accessibility of these sensors, paving the way for their broader adoption.

Future developments are expected to focus on improving sensor durability, reducing costs, and expanding the range of applications. The integration of AI-driven data processing, wireless communication, and microfluidic systems is anticipated to further boost efficiency and usability. As these technologies continue to evolve, optical fiber pH sensors are poised to play a critical role in advancing precision measurement systems, ensuring accurate and reliable pH detection in complex and dynamic environments.

By overcoming the current limitations and leveraging State-of-the-Art innovations, optical fiber sensors have the potential to redefine pH measurement, offering unparalleled accuracy, versatility, and efficiency. This progress is set to drive significant advancements in areas such as healthcare, environmental sustainability, and industrial process optimization, solidifying these sensors’ role as the future standard for pH monitoring technology.

In the authors’ view, optical fiber pH sensors are poised to play a transformative role in several high-impact areas. These include real-time biomedical diagnostics—where miniaturization, electromagnetic immunity, and biocompatibility are essential—as well as environmental monitoring in remote or harsh conditions that challenge traditional electrochemical sensors. Moreover, optical systems offer significant advantages for distributed sensing in industrial process control, particularly in corrosive or high-temperature environments. In the near future, optical fiber sensors are expected to complement or even replace electrochemical sensors in applications requiring long-term stability, remote operation, or integration with advanced platforms such as wearable devices, microfluidic systems, and wireless sensor networks.

## Figures and Tables

**Table 1:** Comparison of pH measurement techniques: cost, advantages, and limitations.

Sensing Method	Key Advantages	Key Limitations	Estimated Cost	Ref.
Glass Electrodes	High accuracy and reliability; sensitive to hydrogen ion concentration; broad pH measurement range (0–14); simple operation; and wide availability.	Fragile and prone to damage; requires frequent calibration and maintenance; and not suitable for harsh or arid environments.	USD 5–21	[9,10,11,30]
Solid-State Electrodes	Robust and resistant to harsh environments; requires minimal maintenance; and suitable for dry or extreme conditions.	Lower sensitivity than glass electrodes; may exhibit reduced accuracy in specific applications.	Low Cost	[12,13,14]
ISFET Electrodes	Durable and breakage-resistant; well suited for portable and compact devices; and performs reliably across diverse environments.	Higher cost than traditional electrodes; requires more complex electronic systems.	High Cost	[15,16,17]
Colorimetric Indicators	Simple to use and inexpensive; does not require complex instrumentation; and suitable for rapid, low-precision measurements.	Limited accuracy; relies on visual estimation or auxiliary analytical tools; and unsuitable for turbid or opaque samples.	Very Low Cost	[18,19,20]
Optical Fiber Sensors	Highly accurate and responsive to subtle pH fluctuations; suitable for harsh and hazardous environments; and ideal for continuous monitoring applications.	Relatively high cost; requires complex setup and routine maintenance.	High Cost	[21,22,23,24]
Fluorescence-Based Techniques	Superior accuracy and high sensitivity; ideal for biomedical and life science applications; and enables fast and continuous measurements.	Requires specialized reagents or sensitive materials; high cost; and susceptible to ambient light and signal interference.	Very High Cost	[25,26,27,28]
Nano-Based Sensors	Very high accuracy and compact size; easily integrated into modern electronic platforms; and suitable for medical and portable applications.	Relatively new technology requiring further validation; high cost; and sensitive to environmental fluctuations and external conditions.	High Cost	[31,32,33]

**Table 3:** Overview of FBG-based pH sensor technologies.

Sensor Configuration and Mechanism	Key Features	Ref.
FBG coated with hydrogel that expands in response to pH changes, inducing axial strain and shifting the Bragg wavelength.	Good linearity (pH 3–7), cost-effective, biodegradable, and repeatable.	[192]
All-polymer FBG with a hydrogel coating that swells under pH variation, producing lateral strain in the polymer structure.	High sensitivity (−0.41 nm/pH) and fast response (30 s).	[197]
Tilted FBG coated with polyaniline (PANI), which alters its optical properties with pH, causing wavelength shifts.	Wide detection range (pH 2–12), temperature-independent, and long-term stability.	[193]
FBG with modeled hydrogel swelling, where a mathematical model estimates the strain induced by hydrogel expansion for accurate pH detection.	Mathematical approach with validated strain analysis for accuracy.	[198]
FBG coated with smart hydrogel materials that respond rapidly to pH fluctuations by changing their volume and inducing strain.	Full pH range (0–14) coverage with enhanced sensitivity and fast response.	[199]
Miniature microfiber FBG fabricated via electrostatic self-assembly technique; pH changes affect its structure and optical response.	Ultra-compact size (~10^−14^ m^3^) with high sensitivity (−72 pm/pH).	[44]
All-polymer FBG with 5–10 μm hydrogel coating that expands/contracts due to pH variations, modulating the Bragg wavelength.	High sensitivity (73 pm/pH) with fast response (<4.5 min).	[200]
FBG with multi-point hydrogel detection, where multiple sensing regions detect pH-induced hydrogel expansion through wavelength shifts.	Experimental validation with ~100 pm shift for pH 4–10 range.	[201]

**Table 4:** Comparison of various FBG-based pH sensors.

pH Range	Sensitivity	Materials Used	Applications	Key Features	Ref.
3–7	12.16 pm/pH	Hydrogel (PVA/PAA)	Acidic pH monitoring	Good repeatability, oscillator behavior	[192]
2–12	46 pm/pH	Polyaniline (PAni)	Biochemical applications, corrosion monitoring	Fast response, biocompatibility	[193]
5–7	73 pm/pH	Hydrogel (PMMA)	Small-scale pH measurement	Fast response (<4.5 min), robust design	[200]
4.66–6.02	117 a.u./pH	Polymers (PDDA/PAA)	Aqueous pH measurement	Fast dynamic response (10 s rise time)	[202]
3–12	79.96 nW/pH	Bromothymol Blue (BTB)	Chemical pH measurement	High sensitivity, temperature compensation	[203]

**Table 5:** Comparison of luminescence lifetime-based pH sensors.

pH Measurement Method	Key Features	Ref.
Luminescence lifetime-based pH sensing using emission lifetime monitoring rather than intensity-based detection.	Demonstrates the advantages of lifetime-based sensors over intensity-based ones, particularly for bioprocess monitoring.	[46]
Ru(II) polypyridyl complexes used as lifetime-responsive luminophores, covalently immobilized for stable, wide-range pH sensing.	Provides enhanced photostability, tunable pKa ranges, and robust performance across the pH range 3.5–8.5.	[213]
FRET mechanisms integrated with luminescence lifetime changes for pH detection.	Offers high accuracy, environmental stability, and minimal interference due to self-referencing decay time signals.	[214]
Microsecond-range luminescence decay pH sensors developed using Ru(II) complexes and pH-sensitive acceptors.	Ensures long-term stability, high sensitivity, and compatibility with frequency–domain detection systems.	[215]

**Table 6:** Summary of research on optical fiber evanescent wave sensors for accurate pH measurements.

Description	Key Topics	Ref.
Study on evanescent wave optical fiber sensing for temperature, humidity, and pH measurement.	Optical fibers with porous silica for stable and sensitive pH detection under temperature variations.	[48]
Evanescent wave-based optical fiber sensors detect changes in the refractive index of the surrounding medium, enabling accurate pH measurement.	High-sensitivity pH measurement based on refractive index changes in the surrounding medium detected through optical fiber transmission.	[229]
Development of an evanescent wave absorption-based fiber-optic biosensor with polyaniline cladding.	Evanescent wave absorption, chemical sensing.	[230]
Investigation of a new fiber-optic sensor configuration utilizing evanescent field absorption for chemical sensing.	Enhanced fiber-optic pH sensor through cladding removal and Fe_3_O_4_@BaMoO_4_: Eu nanocoating to improve measurement accuracy.	[231]
Implementation of a coil-shaped plastic optical fiber sensor for pH and concentration response analysis.	Coil-shaped optical fiber, pH, and concentration response.	[85]

**Table 7:** Comparison of pH measurement performance of various sensor types.

Sensor Type	pH Range	Sensitivity	Response Time	Ref.
Fluorescence-based	1.6–13.2	~0.02 pH units	~5–10 s	[133]
Absorbance-based	4–10 (up to 3–11)	~0.44 nm/pH	~10 s	[37]
SPR-based	2–12 (up to 14)	~0.01 pH resolution	Seconds to minutes	[157]
Interferometric	1.95–11.89	~11 nm/pH [41]	~1.6–15.7 s	[41]
FBG-based	2–12	12–117 pm/pH	10–30 s	[192,193]
Lifetime-based	3–10	~0.01–0.05 pH	A few seconds—3 min	[221]
Evanescent wave-based (OFEWSs)	2–12	Variable (e.g., ~−27.8 pm/ppm)	Seconds	[85,233,236]

## Data Availability

No new data were created or analyzed in this study.
